# Inducing β Phase Crystallinity in Block Copolymers of Vinylidene Fluoride with Methyl Methacrylate or Styrene

**DOI:** 10.3390/polym9080306

**Published:** 2017-07-26

**Authors:** Nahal Golzari, Jörg Adams, Sabine Beuermann

**Affiliations:** 1 Institute of Technical Chemistry, Clausthal University of Technology, Arnold-Sommerfeld-Strasse 4, 38678 Clausthal-Zellerfeld, Germany; nahal.golzari@tu-clausthal.de; 2 Institute of Physical Chemistry, Clausthal University of Technology, Arnold-Sommerfeld-Strasse 4, 38678 Clausthal-Zellerfeld, Germany; adams@pc.tu-clausthal.de

**Keywords:** fluoropolymers, vinylidene fluoride, block copolymers, crystallization, phase separation

## Abstract

Block copolymers of poly(vinylidene fluoride) (PVDF) with either styrene or methyl methacrylate (MMA) were synthesized and analyzed with respect to the type of the crystalline phase occurring. PVDF with iodine end groups (PVDF-I) was prepared by iodine transfer polymerization either in solution with supercritical CO_2_ or in emulsion. To activate all iodine end groups Mn_2_(CO)_10_ is employed. Upon UV irradiation Mn(CO)_5_ radicals are obtained, which abstract iodine from PVDF-I generating PVDF radicals. Subsequent polymerization with styrene or methyl methacrylate (MMA) yields block copolymers. Size exclusion chromatography and NMR results prove that the entire PVDF-I is converted. XRD, FT-IR, and differential scanning calorimetry (DSC) analyses allow for the identification of crystal phase transformation. It is clearly shown that the original α crystalline phase of PVDF-I is changed to the β crystalline phase in case of the block copolymers. For ratios of the VDF block length to the MMA block length ranging from 1.4 to 5 only β phase material was detected.

## 1. Introduction

Most hydrofluorocarbon polymers show extraordinary thermal stability, chemical inertness, and weatherability, as well as being stable against various types of radiation. Large scale applications encompass, e.g., coatings, membranes, tubes, and piping equipment [1,2] Poly(vinylidene fluoride) (PVDF) is special, because additionally it shows ferro-, piezo-, and pyroelectric properties [3,4,5,6,7,8,9,10]. These electroactive properties are associated with the all trans conformation β crystalline phase of PVDF. However, the semi-crystalline polymer forms not only β phase material, but four more polymorphs (α, γ, δ, and ε phase) which are known [11,12].

The α polymorph is the most readily available phase. Several processes were reported to obtain β phase material, such as ultrafast cooling, melting under high pressure, mechanical stretching of the α polymorph, and blending with poly(methyl methacrylate) (PMMA), BaTiO_3_ or palladium. An excellent overview on the various phases of PVDF and applications as electroactive materials is presented in reference [13]. In addition to the homopolymer of vinylidene fluoride (VDF), in binary and ternary copolymers of VDF with trifluoroethylene and chlorotrifluoroethylene β phase formation is found [3,14,15]. Due to higher sterical hindrance compared to VDF homopolymers the formation of the all trans β phase is preferred [13].

Frequently, blends of PVDF and PMMA are considered for β phase formation [16] due to favorable interactions between the highly electronegative F atoms in PVDF and the carbonyl groups of PMMA. Moreover, steric reasons are supposed to favor the trans conformation of both polymers and may lead to a chain extension and consequently β crystallinity is promoted [16]. In addition, the influence of the glass transition temperature, *T*_g_, on the relative growth rates of α and β spherulites is discussed [16]. Depending on polymer molecular weights and the ratio of both polymers in the blend, phase separation may occur. If phase separation in polymer blends induces β phase crystallinity of the PVDF domains, it appears particularly interesting to consider block copolymers consisting of one PVDF block. Again, phase separation is expected to occur and β phase crystallinity should occur.

So far the number of reports on PVDF block copolymers is still rather small. Reversible deactivation transfer polymerizations frequently used for block copolymer synthesis of styrene and many (meth)acrylate monomers are difficult to be performed with VDF, mainly due to the highly reactive primary propagating radical. Methods involving degenerative chain transfer constitute an exception from the above said. Iodine transfer polymerizations (ITP) [17,18,19,20], the use of xanthates [21,22,23,24] or reversible addition fragmentation transfer (RAFT) polymerizations [25,26] allow for good control of molecular weights, result in low dispersities, and may yield block copolymers.

Generally, in ITP perfluorinated alkyl iodides such as C_6_F_13_I or C_6_F_12_I_2_ are used as chain transfer agents [19,20]. Due to the rather low bond energy between iodine and the propagating chain, the transfer reaction of iodine is reversible. Regular head to tail addition leads to –(CF_2_–CH_2_)– motifs in the polymer chain. Further, tail to tail, tail to head, and head to head additions play an important role in VDF polymerizations leading to –CF_2_–CH_2_–CH_2_–CF_2_– and –CH_2_–CF_2_–CF_2_–CH_2_– sequences in the polymer chains as well as –CH_2_–CF_2_–I and –CF_2_–CH_2_–I end groups as identified by NMR spectroscopy. –CH_2_–CF_2_–I is at least 25 times more active than –CF_2_–CH_2_–I [27]. As a consequence, the fraction of inactive –CF_2_–CH_2_–I chain ends increases with conversion. Previously, the active species, –CH_2_–CF_2_–I, was used for follow-up reactions [28,29,30]. In all cases, only the polymer with active end groups was used.

Asandei and coworkers reported a synthetic strategy using Mn_2_(CO)_10_ for activation of both iodine polymer end groups, consequently allowing for an efficient transformation of all polymer chains [31,32,33]. Since the linkage between two Mn atoms is weak (20–40 kJ) [34,35], visible light allows for the production of two ^•^Mn(CO)_5_ metalloradicals with good quantum efficiency [36]. ^•^Mn(CO)_5_ radicals being very good halide abstractors irreversibly activate both iodine end groups originating from ITP and Mn(CO)_5_–I is formed [31]. Both radicals obtained (–CF_2_^•^ and –CH_2_^•^) may react with an added monomer to initiate a free radical polymerization leading to block copolymers where PVDF-I serves as a macroinitiator.

A different synthetic strategy is the preparation of block copolymers via functional benzoyl peroxide initiated polymerization of VDF and subsequent synthesis of the second block using atom transfer radical polymerization or ring opening polymerization [37,38,39]. For example, the resulting block copolymers with the second block being poly(butyl methacrylate) showed β phase crystallinity of the PVDF block with a molecular weight of around 15,000 g·mol^−1^ and a PVDF molar content of around 0.5.

Due to the above mentioned favorable interactions and the associated miscibility of PMMA and PVDF, in this contribution the synthesis of block copolymers of VDF with MMA is reported. In addition, some copolymers with styrene as the second monomer were prepared. Firstly, PVDF-I was synthesized by ITP in supercritical carbon dioxide as solvent [40,41] or in emulsion [42]. Then, with PVDF-I as macroinitiator Mn_2_(CO)_10_-photomediated free radical polymerization with MMA or styrene were carried out to yield well-defined block copolymers. The molecular weight of the initial PVDF macroinitiator and the ratio of the block lengths was varied. Atomic Force Microscopy (AFM) of thin films is considered to identify phase separation of both polymer blocks. In order to identify whether the PVDF domains are crystalline and to analyze which type of crystalline phase is determined, Fourier transform infrared spectroscopy (FTIR), X-ray powder diffraction (XRD), and differential scanning calorimetry (DSC) were used.

## 2. Methods and Materials

### 2.1. Materials

Vinylidene fluoride (VDF, Dyneon GmbH, Burgkirchen a.d.Alz, Germany, 99.5%), styrene (S, Sigma-Aldrich, Taufkirchen, Germany, 99.5%), methyl methacrylate (MMA, Sigma-Aldrich, Taufkirchen, Germany, 99%), di-*tert*-butylperoxide (DTBP, Merck, Darmstadt, Germany, 98%), 1-iodoperfluorohexane (ABCR, Karlsruhe, Germany, 99%), 1,4-diiodooctafluorobutane (Dyneon GmbH, Burgkirchen a.d.Alz, Germany), dimanganese decacarbonyl (Sigma-Aldrich, Taufkirchen, Germany, 98%), methanol (95%), hydrochloric acid (37%), *N*,*N*-dimethyl acetamide (DMAc, Acros, Geel, Belgium, 99%), ammonium 4,8-dioxa-3*H*-perfluorononanoate (Dyneon, GmbH, Burgkirchen a.d.Alz, Germany), ammonium peroxydisulfate (Fluka, Honeywell, Hannover, Germany, ≥98.0%), carbon dioxide (Air Liquide, Paris, France, 99.8%), *N,N*-dimethylformamide-d_7_ (Deutero GmbH, Kastellaun, Germany, 99.5%), and acetone-d_6_ (Deutero GmbH, Kastellaun, Germany, 99.8%) were used as received.

### 2.2. Characterization

To characterize the polymers, the following techniques and equipment were used. Size-exclusion chromatography (SEC) measurements were carried out at a column temperature of 45 °C using DMAc, which contains 0.1% LiBr as eluent. The SEC set-up consists of an Agilent 1200 isocratic pump, an Agilent 1200 refractive index detector, and four PSS GRAM columns (Guard, 100 Å, 3000 Å, and 3000 Å) from Polymer Standard Service (PSS). Measurements were carried out at a flow rate of 1 mL·min^−1^. Polystyrene standards (PSS) were used for calibration. For FT-IR measurements a Vertex 70 Bruker spectrometer (Bruker Optik GmbH, Bremen, Germany) equipped with a globar source and a photoacoustic cell (PA301) was used. Spectra were measured with a resolution of 4 cm^−1^. ^1^H and ^19^F NMR spectra of the polymers were recorded on a Bruker AVANCE 400 MHz spectrometer at room temperature. Acetone-d_6_ and also *N,N*-dimethylformamide-d_7_ were used as solvents. To characterize the phase separation of the copolymers, thin polymer films were prepared either by casting a solution directly onto clean mica or by spin coating onto the same substrate (5 mg polymer in 1 mL DMAc, spin-coater WS-650MZ, Laurell, North Wales, PA, USA). These films were analyzed with an AFM (extended multimode, NanoScope IIIa controller, Veeco/Digital Instruments, Plainview, NY, USA) operating in tapping mode at room temperature in air. To selectively etch the PMMA or PS blocks while keeping the PVDF intact, air-plasma generated in a RF plasma cleaner (PDC-32G, Harrick-Plasma, Ithaca, NY, USA) was used for up to 60 s. This technique has been used successfully to contrast PS in PMMA-PS block copolymers [43]. XRD analyses at KIT were conducted by a STADI MP diffractometer (STOE, Darmstadt, Germany) with Ge-monochromatized Cu–Kα radiation (λ = 1.54060 Å). The XRD measurements at TUC were conducted with Cu–Kα (graphite monochromator) as well. A Bruker AXS D8 Discover diffractometer was used, equipped with a General Area Diffraction System (GADDS, Bremen, Germany) as detector. DSC measurements were performed with a DSC 1/500658/200W STARe system by Mettler Toledo, Columbus, OH, USA. This system is equipped with a FRS5 sensor and liquid nitrogen cooling. Each sample passes through a complete heating and cooling cycle before the second heating run is used for analysis. The heating or cooling rate is 10 °C·min^−1^ for all measurements.

### 2.3. Synthesis of PVDF-I

Polymers with iodine end group (PVDF-I) were synthesized by iodine transfer polymerization (ITP). C_6_F_13_I was used as the chain transfer agent, DTBP as the initiator, and CO_2_ as the solvent. A typical experiment was performed at a constant temperature of 120 °C and an initial pressure of 1500 bar. During polymerization the pressure decreased to around 850 bar due to volume contraction upon consumption of the gaseous monomer. To produce PVDF homopolymers with a number average molecular weight, *M*_n_, between 1500 and 2500 g·mol^−1^, a weight fraction of VDF of around 70%, 1.5 g DTBP (10.4 mmol, *c* = 0.076 mol·L^−1^), and 8.0 g C_6_F_13_I (1.8 mmol, *c* = 0.13 mol·L^−1^) were used. PVDF-I with *M*_n_ > 10^4^ g·mol^−1^ was obtained from reactions with reduced quantities of initiator and chain transfer agent: 0.75 g DTBP (5.1 mmol, *c* = 0.056 mol·L^−1^) and 4.0 g C_6_F_13_I (9.0 mmol, *c* = 0.097 mol·L^−1^) were used. The preparation of the reaction mixture, the polymerization procedure, and the reaction set-up were detailed elsewhere [40].

I-PVDF-I used for samples 2 and 3 is synthesized by emulsion polymerization. The reaction was performed at 90 °C and 15 bar for 4 h. Ammonium 4,8-dioxa-3*H*-perfluorononanoate was used as the surfactant (0.022 mol·L^−1^), ammonium peroxydisulfate as the initiator (6 mmol·L^−1^) and C_4_F_8_I_2_ as the chain transfer agent (12 mmol·L^−1^) [42].

The number average molecular weights *M*_n_ and dispersities of the PVDF homopolymers are listed in Table 1. It should be noted that *M*_n_ data refers to SEC calibration with polystyrene standards. In order to estimate absolute molecular weights the principle of universal calibration was applied [44]. With Mark-Houwink parameters *K* and *a* being known for polystyrene and for PVDF in dimethyl acetamide as eluent [45], *M*_n_ values are derived, which are about 15% lower than the *M*_n_ values listed in Table 1. Since the *K* and *a* values were derived from a higher molecular weight sample, and since SEC is generally considered to be associated with an uncertainty of 10% to 15%, we decided on listing the data derived from the primary experimental data.

### 2.4. Synthesis of the Block Copolymers (PVDF-b-PMMA and PVDF-b-PS)

In a round-bottom flask, 100 mg of PVDF-I (or I-PVDF-I), 1 mL of the other monomer (MMA, 9.4 mmol or styrene 9.1 mmol) and 36 mg of Mn_2_(CO)_10_ (0.092 mmol) were dissolved in 2 mL DMAc. In addition, reactions with different amounts of the monomers (see Table 1) were carried out. The mixture was purged with N_2_ for 10 min, then placed in an oil-bath, and stirred at 90 °C under visible light irradiation (Oriel 60006 lamp, LOT group, Darmstadt, Germany) for 1 h. As suggested by Asandei and coworkers [31], the polymer was precipitated in acidic methanol, filtered, and dried.

## 3. Results and Discussion

ITP of VDF with perfluorinated alkyl iodides serving as chain transfer agents lead to polymers with the following two end groups: –CF_2_–CH_2_–I and –CH_2_–CF_2_–I [27]. As reported by Asandei and coworkers, the ^•^M_n_(CO)_5_ radical obtained upon UV irradiation of Mn_2_(CO)_10_ may abstract I from both chain ends [31]. In the following the PVDF chain extension and block copolymer synthesis based on the use of Mn_2_(CO)_10_ is described.

### 3.1. Chain Extension of PVDF

For block copolymer synthesis PVDF samples with different molecular weights were used. In addition, the amount of macroinitiator and comonomer were varied. Details are displayed in Table 1. In every case, 36 mg of Mn_2_(CO)_10_, 2 mL of DMAc and a reaction time of 1 h were chosen. The results of PVDF macroinitiator and copolymer SEC analyses as well as copolymer compositions derived from NMR analyses are also listed in Table 1.

Samples 2 and 3 were obtained with identical amounts of macroinitiator I-PVDF-I and different amounts of MMA. As expected, the higher MMA concentration leads to significantly higher block copolymer molecular weights. Samples 1, 4, and 5 show that variation of PVDF molecular weight at otherwise identical conditions leads to block copolymers with significantly enhanced molecular weights, while dispersities are slightly lower than for the macroinitiator.

**Figure 1:**
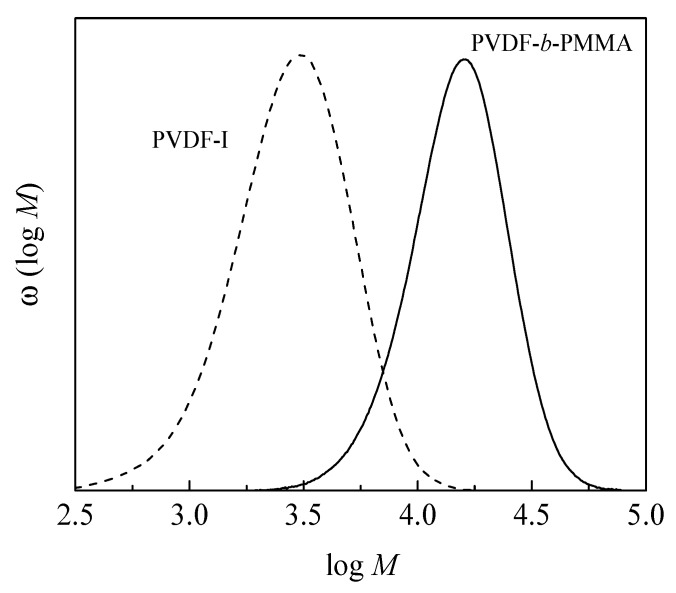
Molecular weight distributions poly(vinylidene fluoride) with iodine end groups (PVDF-I). PVDF-I (dashed) and resulting block copolymer with methyl methacrylate (MMA) (full, sample 1).

In order to test for the formation of block copolymers the molecular weight distributions (MWDs) are considered. While SEC does not give any information on the absolute molecular weights and block lengths due to calibration relative to polystyrene, the position of the MWDs provides information on the chain extension. As an example, Figure 1 gives the MWDs of the macroinitiator and a copolymer containing PMMA as the second block. The block copolymer MWD is clearly shifted to higher molecular weight compared to the MWD of the PVDF-I macroinitiator, which indicates a successful chain extension.

**Figure 2:**
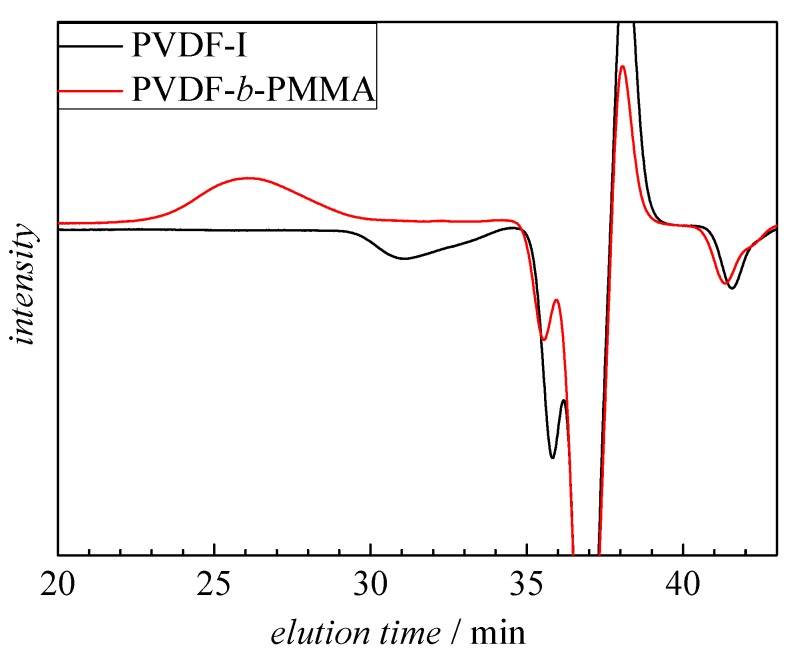
Elution curve of PVDF-I with *M*_n_ = 2033 g·mol^−1^ and copolymer sample 1.

SEC elution chromatograms were analyzed to evaluate whether the entire PVDF-I macroinitiator was transformed. As an example, Figure 2 gives the SEC elution curves for PVDF-I with *M*_n_ = 2033 g·mol^−1^ and the block copolymer sample 1. The negative peak assigned to PVDF-I occurs at an elution time of 31 min, whereas the chromatogram of the block copolymer does not show any contributions from PVDF-I at 31 min. The elution curves of all other copolymers listed in Table 1 show no peak originating from PVDF-I. Contrary to reference [31] where a reaction time of 5 h and a temperature of 110 °C were selected, here 1 h and 90 °C were sufficient for complete conversion of the macroinitiator. The difference is suggested to be due to differences in UV irradiation and consequently differences in the generation of ^•^Mn(CO)_5_ radicals.

### 3.2. Block Copolymer Composition

**Figure 3:**
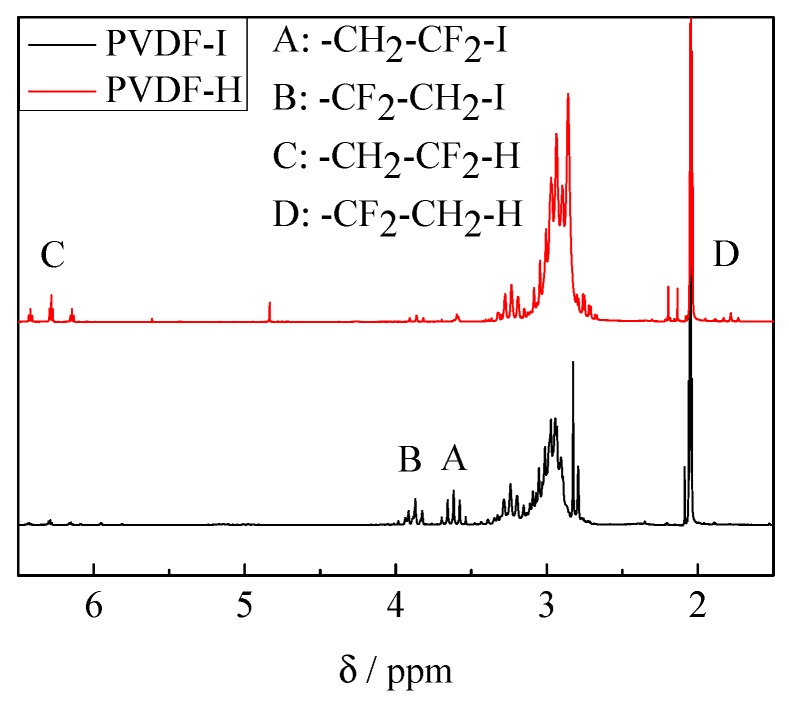
^1^H NMR spectra of PVDF-I (black) and PVDF-H (red).

Since the dispersities of PVDF-I and the block copolymer are rather close, it is anticipated that both chain ends, the CF_2_–I and the CH_2_–I end group, were transformed. To prove whether both end groups were activated and polymerized, end group analyses were carried out via ^1^H and ^19^F NMR spectroscopy. Firstly, a blank reaction of PVDF-I and Mn_2_(CO)_10_ in the absence of comonomer is considered. The reaction was performed with 100 mg PVDF-I dissolved in 3 mL DMAc containing 36 mg of Mn_2_(CO)_10_ (0.092 mmol) under UV irradiation at 40 °C for 3 h. The PVDF radical formed was expected to abstract an H atom either from the solvent or from the polymer. Figure 3 shows ^1^H NMR spectra of the original material (denoted PVDF-I) and PVDF obtained from the blank reaction (denoted PVDF-H). As expected PVDF-I shows both iodine end groups. According to reference [20] the peak at ~3.6 ppm is assigned to –CH_2_–CF_2_–I and the peak at around 3.8 ppm refers to –CF_2_–CH_2_–I. Both peaks are only very weak in the spectrum of PVDF–H. Instead, a peak at about 6.3 ppm is found, which refers to –CH_2_–CF_2_–H being due to transfer to solvent or polymer [46]. The red spectrum referring to PVDF–H also shows a peak at about 1.75 ppm, which represents –CF_2_–CH_3_ originating from –CF_2_–CH_2_–I after replacing I with H [46]. The single peak at δ ~ 4.55 ppm indicates the presence of –CH_2_–CF_2_–CH=CF_2_ [31], which originates from termination via disproportionation. Peaks at δ ~ 2.19 and 2.13 ppm represent the –CH_2_–CF_2_–CH=CF_2_ motif. The strong peak at δ ~ 2.8–3.0 ppm in both spectra refers to the –[CH_2_–CF_2_]*_n_*– head to tail PVDF sequence. The –CF_2_–CH_2_–CH_2_–CF_2_– head to head PVDF sequence is associated with a peak at δ ~ 2.4 which is however hardly seen in both spectra due to the very low *M*_n_ and consequently a small quantity of this sequence in the material. The acetone peak is seen around 2.05 ppm [46,47].

**Figure 4:**
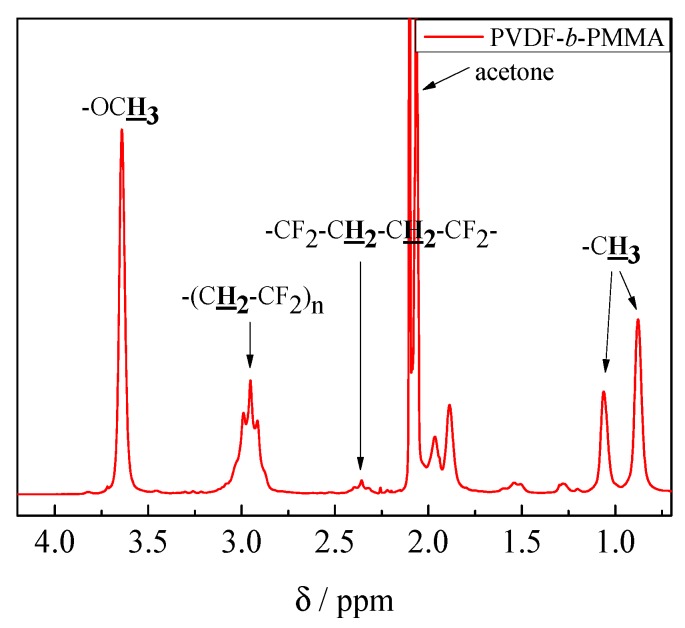
^1^H NMR spectrum of block copolymer sample 5.

The ^1^H NMR spectrum in Figure 4 recorded for a block copolymer of VDF and MMA shows the expected peaks. The very strong peak at about 3.6 ppm is assigned to the –OCH_3_ group of PMMA and two strong peaks between 0.8 and 1.2 ppm to the –CH_3_ group at the main chain of PMMA. In addition, the above-mentioned peaks assigned to the methylene group of PVDF at 2.4 and 3.0 ppm are seen. Integration of the peak at δ ~ 3.6 ppm for PMMA and at δ ~ 2.4 and 3.0 ppm for PVDF allows for the calculation of the ratio of block lengths according to Equation (1).

(1) $$\frac{n_{\mathrm{PMMA}}}{n_{\mathrm{PVDF}}}=\frac{\frac{1}{3}\int {\mathrm{CH}}_{3}(3.6\;\mathrm{ppm})}{\frac{1}{2}\int {\mathrm{CH}}_{2}(3.0\;\mathrm{ppm})+\frac{1}{2}\int {\mathrm{CH}}_{2}(2.4\;\mathrm{ppm})}$$

The example in Figure 4 represents a block copolymer with *n*_PVDF_ to *n*_PMMA_ of 1 to 0.67 (sample 5). The results for all block copolymers are given in Table 1.

**Figure 5:**
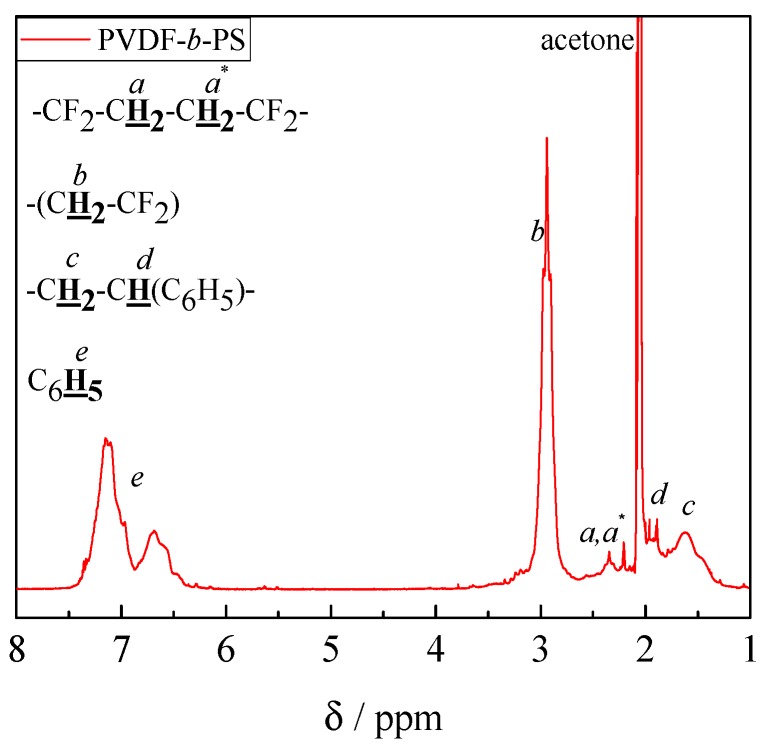
^1^H NMR spectrum of block copolymer sample 9.

The ^1^H–NMR spectrum in Figure 5 recorded for a block copolymer consisting of VDF and styrene shows also the expected peaks. The strong peaks at around 6.6 to 7.2 ppm are assigned to the –C_6_H_5_ group of PS. Two other peaks at 1.94 ppm (–CH_2_–CH–(C_6_H_5_)–) and 1.63 ppm (–CH_2_–CH–(C_6_H_5_)–) also belong to PS. In addition, the peaks at 3.0 and 2.4 ppm assigned to the methylene groups in PVDF are seen. Integration of the peaks at 6.6 to 7.2 ppm for PS and at 2.4 and 3.0 ppm for PVDF allows for the calculation of the ratio of block lengths according to Equation (2).

(2) $$\frac{n_{\mathrm{PS}}}{n_{\mathrm{PVDF}}}=\frac{\frac{1}{5}\int C_{6}H_{5}(6.6\;\mathrm{to}\;7.2\;\mathrm{ppm})}{\frac{1}{2}\int {\mathrm{CH}}_{2}(3.0\;\mathrm{ppm})+\frac{1}{2}\int {\mathrm{CH}}_{2}(2.4\;\mathrm{ppm})}$$

The example in Figure 5 represents a block copolymer with *n*_PVDF_ to *n*_PS_ of 1 to 0.56. The results for all block copolymers containing styrene are listed in Table 1.

The ^19^F–NMR results also show that –CH_2_–CF_2_–I and –CF_2_–CH_2_–I chain ends of the PVDF macroinitiator were activated and polymerized. Peaks at δ ~ −38.4 ppm and δ ~ –108 ppm referring to –CH_2_–CF_2_–I and –CF_2_–CH_2_–I [31,47] respectively, of the macroinitiator clearly disappeared after copolymerization (see Figure S1 in Supporting Information).

### 3.3. Crystallinity of the Block Copolymers

**Figure 6:**
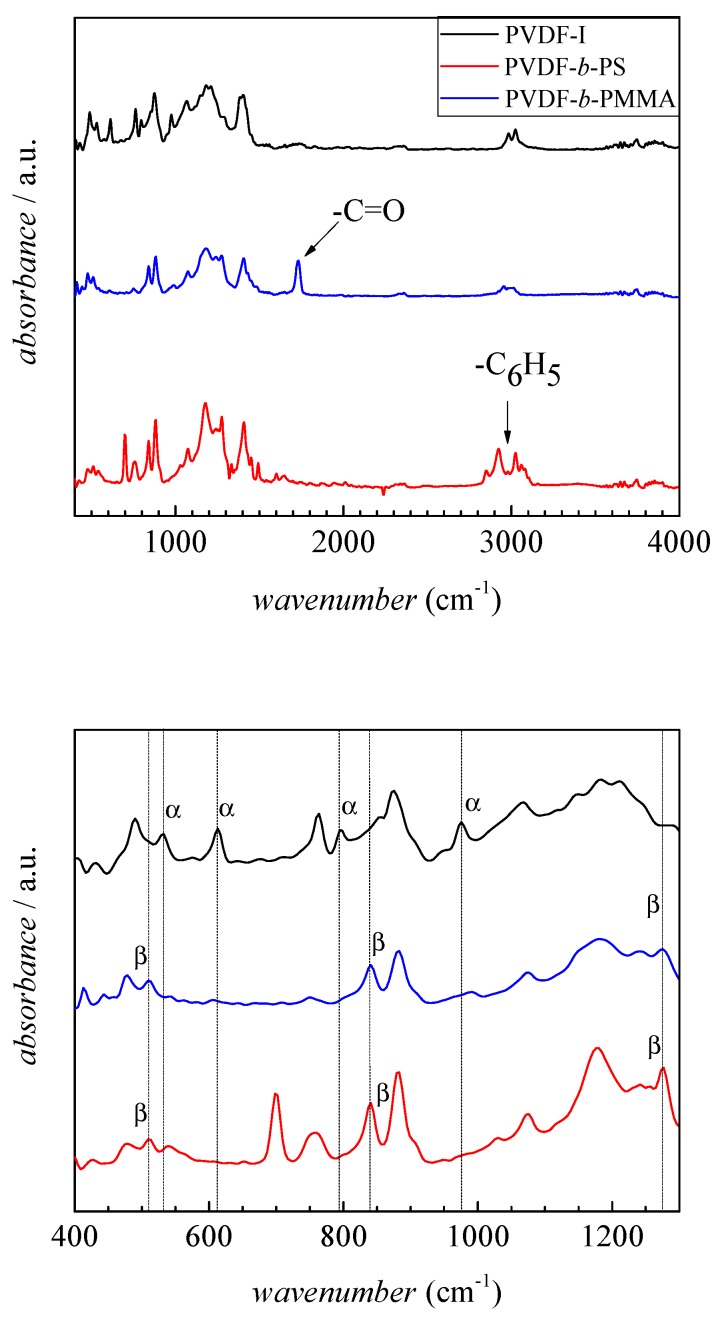
FT-IR results of PVDF-I (black), PVDF-*b*-PMMA (blue, sample 1), and PVDF-*b*-PS (red, sample 9). The vertical lines indicate peaks that are representative of either the α or β crystalline phase of PVDF.

To obtain additional information on the crystallinity of the products, FT-IR spectra were recorded. The FT-IR spectra of PVDF homopolymer and the block copolymers are given in Figure 6. One of the most prominent difference is the strong band at 1730 cm^−1^, which is assigned to the carbonyl group in PMMA (blue spectrum) which is absent in the PVDF spectrum. In addition, the IR spectrum of the PMMA block copolymer shows a broad peak at 2951 cm^−1^ assigned to –OCH_3_ of PMMA. The spectrum of the block copolymer with PS (red spectrum) shows the aromatic C–H stretching vibrations at 2850, 2923, 3025, and 3060 cm^−1^. The peaks at 1493 and 1062 cm^−1^ are assigned to the aromatic C–C bond stretching vibration. The absorbances at 3000 and 3100 cm^−1^ represent the C–C vibrations and peaks at 1151 cm^−1^, as well as at 1193 cm^−1^ for C–F vibrations of the PVDF block [30,48]. The spectra clearly indicate the presence of both monomer units in the products.

To obtain information on the crystallinity of the PVDF block, the enlarged spectra in the wavenumber range from 500 to 1300 cm^−1^ depicted in the lower part of Figure 6 are considered. The spectrum of the PVDF-I macroinitiator shows distinct peaks at 532, 614, 795, and 976 cm^−1^ indicating the presence of α phase PVDF. On the contrary, the spectra of the block copolymers show none of the above mentioned peaks. New peaks at 510, 841, and 1276 cm^−1^ occur, which suggest the presence of β crystalline PVDF domains in the copolymer. Thus, the formation of PVDF-*b*-PMMA or PVDF-*b*-PS is suggested to be associated with a change in crystallinity of the PVDF segments.

**Figure 7:**
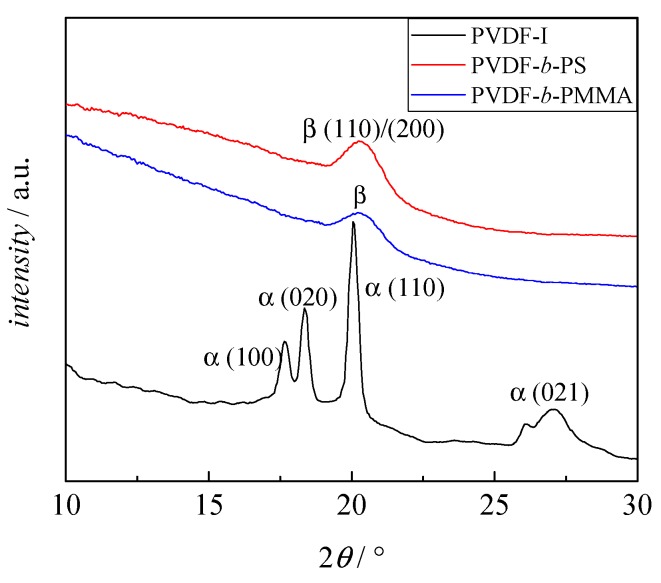
XRD results of PVDF-I (black), block copolymer with MMA (sample 4, blue) and block copolymer with styrene (sample 11, red).

The peak at 841 cm^−1^ in the IR spectrum may also be indicative of γ phase material, however, peaks at 776, 812, 833, and 1234 cm^−1^ also typical for the γ phase are not seen. As pointed out in literature, the flawless identification of β phase PVDF requires additional analyses such as XRD [13]. The results from XRD of PVDF-I and two block copolymers (sample 1 and 9 in Table 1) are depicted in Figure 7. The spectra are clearly different. The PVDF-I curve shows peaks at 2θ values of 17.66°, 18.30°, 19.90°, and 26.56°, which are related to the α phase [13]. On the other hand for the block copolymers only one peak with a maximum at around 20.26° is observed, which indicates the presence of β phase material. Peaks typical for the γ phase at 2θ values of 18.50°, 19.20° or 20.04° are not found [13]. Thus, the results from FT-IR and XRD strongly suggest the transformation of α to β phase PVDF domains in the copolymer. In addition, DSC analyses yield melting temperatures of 165 °C for the block copolymers and 172 °C for the PVDF macroinitiator. These temperatures are characteristic for α and β phase material. Since γ phase PVDF is associated with melting temperatures between 180 and 190 °C [13] the presence of γ phase PVDF can be excluded.

The results demonstrate that the crystal structure of PVDF moieties is transformed from α to β after block copolymer synthesis. To identify which ratio of PVDF block length, *n*_PVDF_, to comonomer block length, *n*_co_, is required for this transformation a number of block copolymers was synthesized. For *n*_VDF_/*n*_co_ ranging from 0.25 to 100 the characteristic IR peaks assigned to β phase material were found. In addition, contributions from α phase were observed. In cases where this ratio was between 1.4 and 5 exclusively the IR peaks indicative of β phase material were detected. With decreasing block length ratio *n*_PVDF_/*n*_co_ the fraction of crystalline material is reduced. In addition to the block length ratio, the absolute lengths of both blocks is important to be considered. The data presented here refer to PVDF segments with lengths between 27 and 153 monomer units. In future, longer PVDF segments need to be used as well.

According to references [16,48,49,50] the miscibility of PVDF and PMMA does not depend on temperature, which is attributed to interactions between the carbonyl group of PMMA and the dipole moment of PVDF as well as hydrogen bonding. Because of steric reasons, the above-mentioned interactions lead to an all trans conformation in both polymers. Further, the presence of MMA units may alter the glass transition temperature of PVDF, which affects the relative growth rates of α and β polymorphs [16].

According to dynamic mechanical measurements, pure PVDF may undergo four relaxations when frequency and temperature are changed [51,52]. One of these relaxations, which is associated with the glass transition may be shifted to higher frequencies and pressures after addition of PMMA. Essentially, PMMA facilitates relaxation from a lower energy level, which may be explained by breaking the interactions and correlations between the PVDF permanent moments in the amorphous-crystalline-interphase and improving the dielectric relaxation possibilities [16].

The piezoelectricity and pyroelectricity of PVDF are associated with the existence of a remnant polarization that is proportional to the degree of crystallinity [16]. PMMA, as the amorphous phase, surrounds individual crystallites, and therefore, affects the degree of crystallinity.

**Figure 8:**
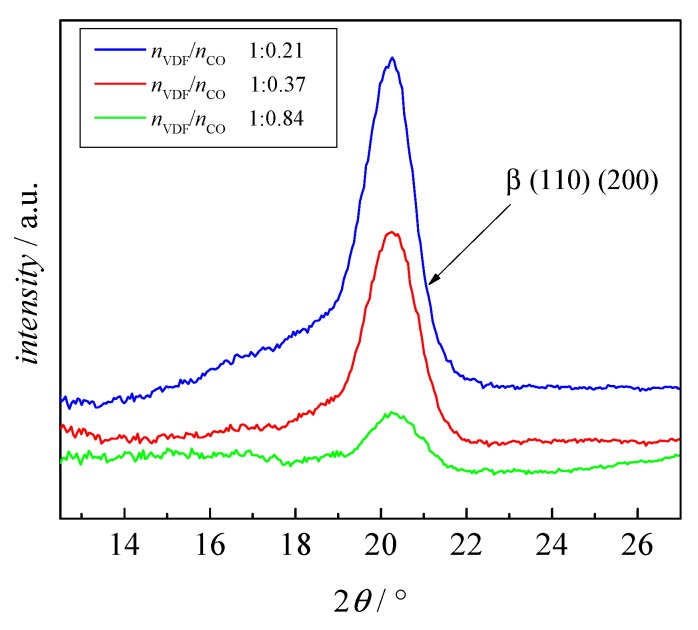
XRD curves for sample 6 (blue), 7 (red), and 8 (green) with the block length ratios as indicated.

Figure 8 compares the XRD results of samples 6, 7, and 8 with PMMA volume fractions of 33%, 46%, and 66%, respectively, in the block copolymer. While it is clearly seen that no significant contributions from α phase are contained, a quantitative comparison of the XRD results of different samples is not feasible. However, the DSC results show that the integration of the melting peak and the degree of crystallinity are proportional to block lengths ratio and volume fraction of PMMA in the block copolymer. In all cases, the integrals of the melting peak and the degree of crystallinity decrease, if the volume fraction of PMMA in the block copolymer increases.

Interestingly the block copolymers with PS as second block also show β phase crystallinity. The reason for this behavior is not yet clear. It may be suspected that the aromatic groups are directing the conformation to an all trans structure of PVDF. For low molecular weight species interactions between F atoms and for example aromatic rings were reported [53,54,55,56].

### 3.4. Phase Separation

**Figure 9:**
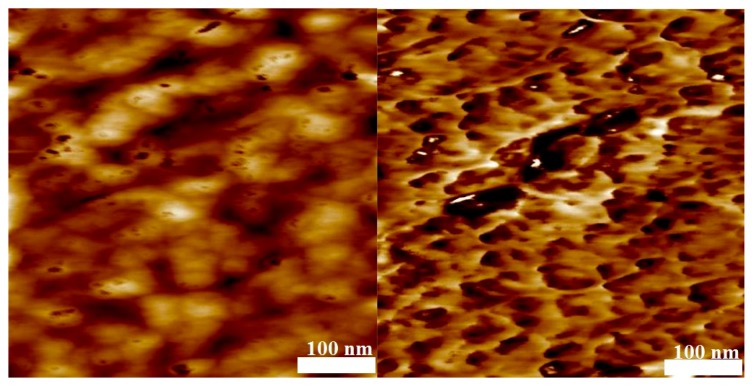
Atomic Force Microscopy (AFM) results of the block copolymer with MMA (sample 6) with *M*_n_ = 12,080 g·mol^−1^ after 10 s of air-plasma treatment. Topography ((**left**) height scale: 5 nm) and phase image ((**right**) phase scale: 90°), Average domain size = 50 nm.

**Figure 10:**
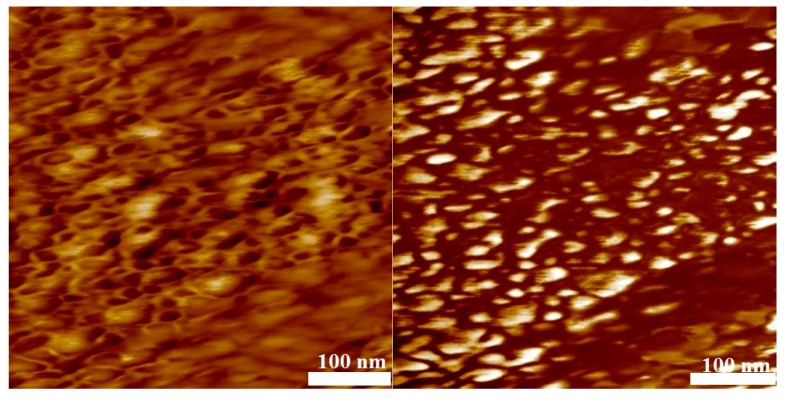
AFM results of the block copolymer with MMA (sample 4, *M*_n_ = 39,450 g·mol^−1^) after 10 s of air plasma treatment. Topography ((**left**) height scale: 10 nm) and phase image ((**right**) phase scale: 50°), average domain size = 75 nm.

**Figure 11:**
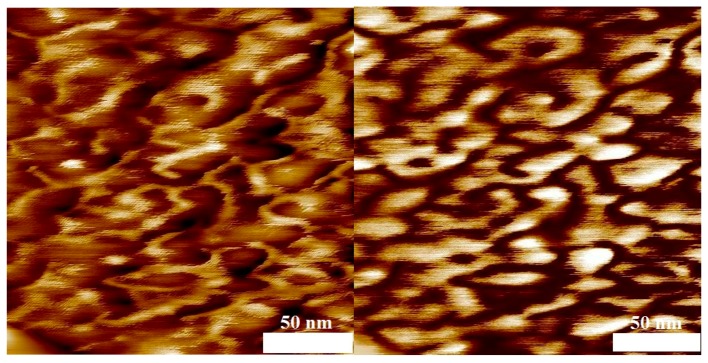
AFM results of the block copolymer with styrene (sample 11, *M*_n_ = 19,856 g·mol^−1^) after 10 s of air-plasma treatment. Topography ((**left**) height scale: 10 nm) and phase image ((**right**) phase scale: 30°), average domain size = 80 nm.

**Figure 12:**
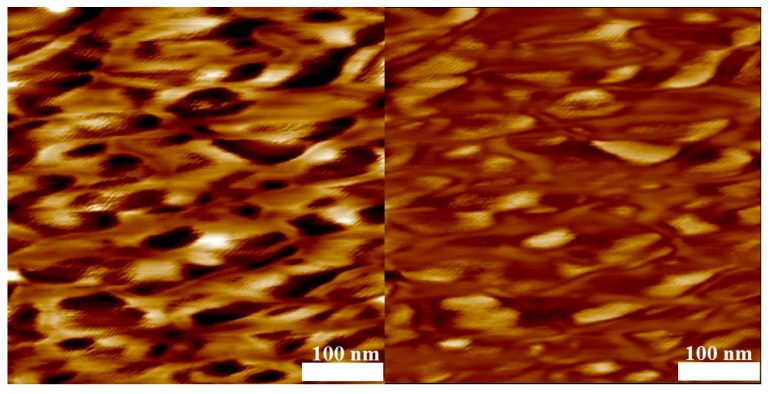
AFM results of the block copolymer with styrene (sample 10, *M*_n_ = 33,040 g·mol^−1^) after 10 s of air-plasma treatment. Topography ((**left**) height scale: 10 nm) and phase image ((**right**) phase scale: 90°), average domain size = 120 nm.

To test for microphase separation of the two polymer blocks, AFM analyses of thin films of samples 4, 6, 10, and 11 obtained by spin coating on mica were carried out. Without selective etching of the PMMA or PS block by air-plasma, flat films were obtained that did not show any phase separation as indicated by the phase-image of the AFM (not shown). The phase-image contrasts in a semi-quantitative way differences in the material properties, e.g., hardness and viscoelasticity. After 10 s of air plasma treatment for every sample, a height variation in the topography image on the left hand side of Figure 9, Figure 10, Figure 11 and Figure 12 is visible and the phase image on the right hand side of these figures shows small domains in a continuous matrix. These domains may be associated with a PVDF-rich microphase and the size of these domains scales with the volume fraction of PVDF in the copolymer. In Figure 9 and Figure 10, it is notable that the continuous matrix in which the PVDF-domains are embedded covers a much smaller surface fraction in sample 6 compared to sample 4. This observation corresponds with the lower PMMA volume fraction of ϕ_co_ = 0.33 in sample 6 compared to ϕ_co_ = 0.85 in sample 4. Control experiments with blends of PMMA and PVDF homopolymers showed no phase separation.

AFM measurements at identical conditions were carried out for two copolymers with styrene blocks of different length (Figure 11 and Figure 12). As expected, the size of the domains are proportional to the PVDF volume fraction in the copolymer. In Figure 12, it is notable that the continuous matrix containing the PVDF–domains covers a smaller surface fraction in sample 10 compared to sample 11 (Figure 11) because of the lower value of ϕ_co_ = 0.50 compared to ϕ_co_ = 0.58, respectively.

## 4. Conclusions

PVDF-I obtained from iodine transfer polymerization served as macroinitiator for MMA and styrene polymerization. Due to four well-known propagation reactions in VDF polymerizations, two types of PVDF end groups were obtained: CF_2_–CH_2_–I and CH_2_–CF_2_–I, which have very different reactivities. Following Asandei et al. the entire polymer material may be functionalized upon UV irradiation of Mn_2_(CO)_10_ in the presence of PVDF-I [31]. ^•^Mn(CO)_5_ radicals obtained due to UV irradiation abstract iodine from both PVDF-I end groups. The resulting PVDF radicals initiate MMA and styrene free radical polymerizations leading to block copolymers of VDF and MMA or styrene, as indicated by SEC analyses and NMR spectra.

FTIR spectra suggest that the crystal phase is transformed from the α phase in case of PVDF-I to β phase PVDF–domains in case of the block copolymers. XRD measurements confirmed the transformation from α to β crystalline phase. Varying block length ratios *n*_VDF_/*n*_co_ from 0.25 to 100 resulted in β phase crystallinity. For ratios from 1.4 to 5 exclusively β phase material was detected. AFM results indicate a phase separation of the PVDF-segments from the other polymer, PMMA or PS. Mixtures of corresponding homopolymers do not show a distinct phase separation in the AFM images. The data indicates that the volume fraction of both monomer units within the block copolymers affects the structure formation.

## Figures and Tables

**Table 1:** Details of block copolymer synthesis and resulting block copolymers properties with the amount of macroinitiator *m*_PVDF_, the volume of comonomer *V*_co_, molecular weights (*M*_n,block_), dispersities (*D*_block_), molar ratio of VDF and comonomer (*n*_VDF_/*n*_co_), and volume fraction of the comonomer in the copolymer. *M*_n,PVDF_ and *D*_PVDF_ refer to the PVDF-I ^a^ or I-PVDF-I ^b^ macroinitiator. The SEC was calibrated with polystyrene. In all cases 36 mg Mn_2_(CO)_10_, 2 mL DMAc and a reaction time of 1 h were chosen.

No.	*M*_n,PVDF_/ g·mol^−1^	*D*_PVDF_	*m*_PVDF_/ mg	Comonomer	*V*_co_/mL	*M*_n,block_/ g·mol^−1^	*D*_block_	*n*_VDF_/*n*_co_	*n*_VDF_	*n*_co_	ϕ_co_
1 ^a^	2033	1.5	100	MMA	1	22,344	1.6	1/3	27	81	0.84
2 ^b^	4344	2.0	100	MMA	0.5	15,176	1.6	1/0.83	58	48	0.64
3 ^b^	4344	2.0	100	MMA	4	40,524	1.8	1/4	58	232	0.87
4 ^a^	4518	1.4	100	MMA	1	39,450	1.8	1/2.3	60	138	0.85
5 ^a^	11,500	1.4	100	MMA	1	50,520	1.7	1/0.67	153	102	0.58
6 ^a^	11,500	1.4	100	MMA	0.2	12,080	1.5	1/0.21	153	33	0.33
7 ^a^	11,500	1.4	100	MMA	0.5	36,170	1.3	1/0.37	153	57	0.46
8 ^a^	11,500	1.4	100	MMA	1.5	60,880	2.1	1/0.84	153	128	0.66
9 ^a^	2033	1.5	100	S	1	13,220	3.6	1/0.56	27	15	0.59
10 ^a^	11,500	1.4	100	S	3	33,040	1.3	1/0.91	153	140	0.58
11 ^a^	11,500	1.4	200	S	1	19,856	1.9	1/0.71	153	109	0.50
12 ^a^	11,500	1.4	200	S	2	21,669	1.7	1/0.77	153	118	0.54

## Supplementary Materials

The following are available online at www.mdpi.com/2073-4360/9/8/306/s1, Figure S1: A comparison of ^19^F NMR spectra of PVDF-I (blue) and the corresponding PVDF-*b*-PMMA (red, sample 1 in Table 1).
