# Supplementary material for: Inducing β Phase Crystallinity in Block Copolymers of Vinylidene Fluoride with Methyl Methacrylate or Styrene
Source: Polymers (Basel). 2017 Jul 26;9(8):306. doi: 10.3390/polym9080306 (PMC6418974; doi:10.3390/polym9080306)
Supplement: Supplementary file 1 [file polymers-09-00306-s001.pdf]

Supplementary Information: Inducing β Phase Crystallinity in Block Copolymers of Vinylidene Fluoride with Methyl Methacrylate or Styrene

Nahal Golzari, Jörg Adams, and Sabine Beuermann

^{1}H−NMR spectra of block copolymers consisting of PVDF and either polystyrene or PMMA are presented in the main text as Figures 3, 4 and 5. In order to monitor the transformation of all PVDF iodine end groups ^{19}F−NMR spectra are particularly valuable. As an example Figure S1 gives a ^{19}F−NMR spectra of PVDF−I and a corresponding block copolymer with PMMA as second block. The assignment of the peaks follows publications by the groups of Ameduri [1,2] and Asandai [1].

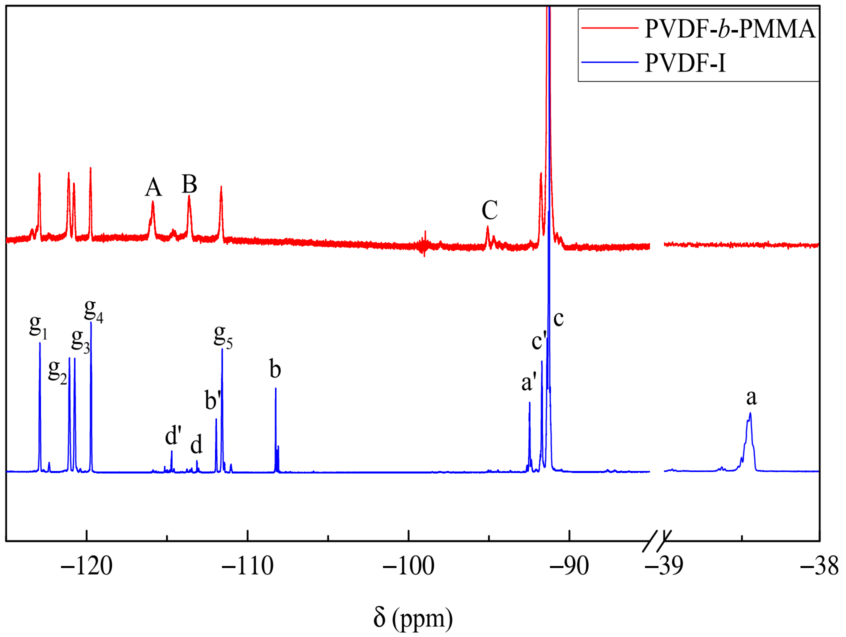

Figure S1. A comparison of ^{19}F−NMR spectra of PVDF−I (blue) and a corresponding block copolymer with PMMA as second block (red, sample 1 in Table 1 of the main text).

The blue spectrum in Figure S1 refers to PVDF-I. The assignment of the iodine chain ends is as follows:

$-CH_2-CF_2-CH_2-CF_2-CH_2-CF_2-I$ (**a**) at δ = −38.5 ppm

$-CH_2-CF_2-CH_2-CF_2-CH_2-CF_2-I$ (**a′**) at δ = −92.5 ppm

$-CH_2-CF_2-CF_2-CH_2-I$ (**b**) at δ = −108.3 ppm

$-CH_2-CF_2-CF_2-CH_2-I$ (**b′**) at δ = −112.0 ppm

Upon block copolymer synthesis all of the above-mentioned peaks disappeared, as indicated by the red spectrum in Figure S1, which confirms that both PVDF chain ends were successfully activated. The peaks assigned to the main chain of PVDF marked with ***c*** and ***c′*** (–C**F**$_2$–[CH$_2$–CF$_2$]$_n$–CH$_2$–) are observed at δ = −91.3 ppm and δ = −91.6 ppm. In addition, ***d*** and ***d′*** assigned to the HH motif (-CH$_2$-C**F**$_2$-CF$_2$-CH$_2$-CH$_2$-CF$_2$- and -CH$_2$-CF$_2$-C**F**$_2$-CH$_2$-CH$_2$-CF$_2$-) are found at δ = −113.1 ppm and at δ = −114.7 ppm in both spectra.

Interestingly, after block copolymer synthesis two new peaks at δ = −115.8 ppm (A) and δ = −113.6 ppm (B) are observed in the red spectrum, which refer to –CH$_2$–C**F**$_2$–CH$_2$–CF$_2$–H and –CH$_2$–C**F**$_2$–C**F**$_2$–CH$_2$–CH$_2$–CF$_2$–H. The H end group is suggested to be due to termination via disproportionation of PVDF radicals after abstraction of iodine. Peak B at δ = −113.6 ppm overlaps with the very small peak ***d*** at δ = −113.1 ppm assigned to the HH sequence. The other interesting peak in the block copolymer spectrum is a small peak at δ = −95 ppm assigned to **C** in the red spectrum. Since the peak does not exist in the PVDF–I spectrum it should be due the linkage between the PVDF and the PMMA block. The peak is suggested to be indicative of –CF$_2$– next to the first MMA unit [1–3].

The C$_6$F$_{13}$ group originating from the chain transfer agent is also clearly seen in both spectra. The various fluorine atoms may be identified as follows:

CF$_3$–C**F**$_2$–C**F**$_2$–C**F**$_2$–C**F**$_2$–C**F**$_2$–[CH$_2$–CF$_2$]$_n$–CH$_2$– (**g**, not shown) at δ = −69.3

C**F**$_3$–CF$_2$–C**F**$_2$–C**F**$_2$–C**F**$_2$–C**F**$_2$–[CH$_2$–CF$_2$]$_n$–CH$_2$– (**g$_1$**) at δ = −122.9 ppm

C**F**$_3$–C**F**$_2$–CF$_2$–C**F**$_2$–C**F**$_2$–C**F**$_2$–[CH$_2$–CF$_2$]$_n$–CH$_2$– (**g$_2$**) at δ = −121.1 ppm

C**F**$_3$–C**F**$_2$–C**F**$_2$–CF$_2$–C**F**$_2$–C**F**$_2$–[CH$_2$–CF$_2$]$_n$–CH$_2$– (**g$_3$**) at δ = −120.7 ppm

C**F**$_3$–C**F**$_2$–C**F**$_2$–C**F**$_2$–CF$_2$–C**F**$_2$–[CH$_2$–CF$_2$]$_n$–CH$_2$– (**g$_4$**) at δ = −119.8 ppm

C**F**$_3$–C**F**$_2$–C**F**$_2$–C**F**$_2$–C**F**$_2$–CF$_2$–[CH$_2$–CF$_2$]$_n$–CH$_2$– (**g$_5$**) at δ = −111.5 ppm

The differences in intensity and chemical shifts observed in both spectra of Figure S1 are due to different concentrations, different frequencies, and the use of a 600 MHz spectrometer for PVDF-I and a 400 MHz spectrometer for the block copolymer.

References

1. Boyer, C.; Valade, D.; Lacroix-Desmazes, P.; Ameduri, B.; Boutevin, B. Kinetics of the Iodine Transfer Polymerization of Vinylidene Fluoride. *J. Polym. Sci. Part A. Polym. Chem.* **2006**, *44*, 5763–5777, doi:10.1002/pola.21654.
2. Balague, J.; Ameduri, B.; Boutevin, B.; Caporiccio, G. Controlled step-wise telomerization of vinylidene fluoride, hexafluoropropene and trifluoroethylene with iodofluorinated transfer agents. *J. Fluorine Chem.* **2000**, *102*, 253-268, doi:10.1016/S0022-1139(99)00287-0.
3. Asandei, A.D.; Adebolu, O.I.; Simpson, C.P. Mild-Temperature Mn$_2$(CO)$_{10}$ Photomediated Controlled Radical Polymerization of Vinylidene Fluoride and Synthesis of Well-Defined Poly(vinylidene fluoride) Block Copolymers. *J. Am. Chem. Soc.* **2012**, *134*, 6080–6083, doi:10.1021/ja300178r.
